# The Dark Side of Green Energy: Glycol Waste and the Microbes That Can Transform It

**DOI:** 10.3390/molecules31101662

**Published:** 2026-05-14

**Authors:** Julia Alicja Dybka, Klaudiusz Tomczyk, Mateusz Szczepańczyk, Katarzyna Ewa Kosiorowska

**Affiliations:** 1 BIOSUS Student’s Scientific Club, Laboratory for Biosustainability, Institute of Biology, Wrocław University of Environmental and Life Sciences, Kożuchowska 5b, 51-631 Wroclaw, Poland; 122810@student.upwr.edu.pl (J.A.D.); klaudiusz.tomczyk@upwr.edu.pl (K.T.); 2 Laboratory for Biosustainability, Institute of Biology, Wrocław University of Environmental and Life Sciences, Kożuchowska 5b, 51-631 Wrocław, Poland; mateusz.szczepanczyk@upwr.edu.pl; 3 Department of Applied Bioeconomy, Wrocław University of Environmental and Life Sciences, 37a, Chełmońskiego Street, 51-630 Wrocław, Poland

**Keywords:** ethylene glycol, propylene glycol, biodegradation, waste management, microbial biotechnology, coolant recycling

## Abstract

The progressive deployment of renewable energy systems has engendered a considerable increase in the generation of glycol-based coolant waste, specifically ethylene glycol (EG) and propylene glycol (PG), thereby raising significant environmental apprehensions. This review analyses the critical environmental challenge and examines the feasibility of microbial degradation as a viable and sustainable alternative to glycol waste treatment, while highlighting significant gaps in current hazardous glycol waste management practices. Present waste management practices are largely founded on incineration or membrane filtration approaches, both of which exhibit significant energy demands and inefficiencies in large-scale waste handling. Reported performance ranges from >99% EG recovery at 10–16 kWh/m^3^ by electrodialysis and 80–95% recovery at 2–4 MJ/kg by vacuum distillation, to ~17 MJ/kg combustion heat from incineration; biological methods, though promising, currently operate below 10% glycol concentration, an order of magnitude below the 10–100% range in real coolants. We analyze the current understanding of metabolic pathways involved in glycol biodegradation, drawing on the peer-reviewed literature, bioinformatics, and patent databases. Special attention is given to the challenges of high glycol concentrations in industrial coolants and the formation of toxic oxidation products during thermal aging. The review also explores recent advances in genetic engineering approaches to enhance microbial degradation efficiency. Finally, we discuss the potential integration of biological recycling methods into existing waste management systems and future prospects for converting glycol waste into value-added products through microbial biotransformation.

## 1. Introduction

The rapid global transition towards renewable energy systems has brought about an unforeseen environmental challenge: an increasing quantities of glycol-based coolants are ending up as waste from solar panels, heat pumps, and photovoltaic installations. In recent years, public awareness has expanded significantly, thereby increasing the demand for unconventional energy sources have also grown. This fact will strengthen the trend toward using installations utilizing glycols as heat exchangers in the upcoming years. Although pure water is a more efficient heat conductor, manufacturers add glycol(s) to coolants to prevent freezing and overheating in solar and geothermal systems. This mixture of glycol(s) and water ensures both a lower freezing point and a higher boiling point than water alone, enabling the systems to operate safely over a wider range of temperatures. Glycol-based heat transfer fluids undergo gradual deterioration under operational conditions, which leads to a significant decrease in their thermal efficiency and overall system performance. These fluids must be replaced at regular intervals specified by the manufacturers to ensure the maximum efficiency of renewable energy systems. Although these cooling systems are crucial for sustainable energy production, they predominantly use ethylene glycol (EG) and propylene glycol (PG) as heat transfer fluids, the regular replacement of which generates large amounts of hazardous waste. The fluids used to fill the system consists of EG and/or PG in various concentrations (10–100%), which is problematic due to the high osmotic pressure that hinders the biological utilization process. Even though, according to Polish standards, propylene glycol (PG) is a low-toxicity compound, the amounts released into the environment so far, primarily into soil, are already treated as contaminants [1]. Ethylene glycol (EG) is highly toxic to humans and animals, as are its decomposition products [2].

The issue of increasing amounts of liquid antifreeze waste, which primarily consists of glycols, is expected to escalate in the near future. This will be influenced, among other things, by the need for changes in the public and commercial sectors, which are in line with the latest European Union regulations (REPowerEU Plan). Given the planned independence from Russia in terms of energy resources, by 2026 all new public and commercial buildings with an area of more than 250 m^2^ will have to be equipped with photovoltaic installations, and existing buildings of this type will be obliged to implement these installations by 2027. The REPowerUE Plan will also cover newly built single-family homes, which will have to be fitted with photovoltaic installations starting in 2029 [3].

**Figure 1:**
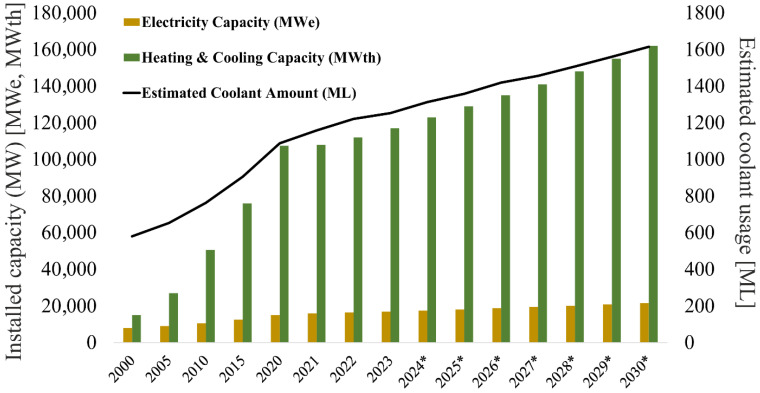
Data on installed electrical capacity (MWe), thermal capacity (MWth) and forecasted growth in the near future (3% projected annual growth). The data has been correlated with the estimated consumption of coolants (Million Liters-ML) between 2000 and 2030, which was/will be necessary to ensure the efficient operation of the installation. The historical data refers to the period from 2000 to 2023, whereas the values for the years 2024 to 2030 are a prognosis (marked with an asterisk *).

Over the past two decades, geothermal energy has shown a constant increase in global use as a renewable source capable of providing both electricity and heat. Globally, installed geothermal electricity capacity has increased from 11 gigawatts (GW) in 2010 to around 15 GW by 2020, reaching 16 GW in 2021, with an average annual growth rate of 3.5%. Nevertheless, despite this growth, the share of geothermal energy in the global installed capacity of renewable energy sources is currently only 0.5%. To achieve the goals of the Paris Agreement on climate change, the International Renewable Energy Agency (IRENA) and the International Geothermal Association (IGA) predict that global installed geothermal capacity must increase significantly, reaching 196.7 GW by 2030 and 872.6 GW by 2050 [3]. In Figure 1, we show the estimated number of glycol-based coolants that have been and will be needed, to ensure the proper functioning of the installed geothermal pumps. All parameters show a steady upward trend, especially thermal power (MWth, megawatts thermal). The estimated consumption of coolants is strongly correlated with the development of the sector, predicted to reach around 1600 million liters (ML) by 2030. The amount of refrigerant filling the ground pipe system of geothermal heat pumps varies depending on the type of refrigerant, system design, soil type, and its thermal properties, as well as pipe length. Nevertheless, based on the available manufacturer data, a closed-loop system using a water–glycol mixture, requires approximately 20 to 120 L per kW (kilowatt) of capacity. A horizontal ground heat exchanger (GHX) typically requires about 35 to 55 m of pipe per kW of heating and cooling capacity [4,5,6]. Based on the available data, we assume that each 4 kW of geothermal installation requires 290 L of coolant (resulting in 72.5 ML per 1000 MW of installed capacity).

Nevertheless, it is important to note that ground geothermal pumps are not the only renewable energy source that uses coolants. This especially true in the context of the current trend towards self-sufficient houses that do not require an external energy source. In such houses, photovoltaic panels are responsible for the power supply, geothermal heat pumps provide heating, and solar panels heat the water. Considering this fact, households will generate large amounts of used, overheated waste coolants containing glycols. Furthermore, due to the current policy of co-financing of such installations, more and more households are deciding to use these alternative forms of energy production. Alongside domestic installations, photovoltaic panels will play a major role in the energy transition and in the consumption of glycol-based coolants.

**Figure 2:**
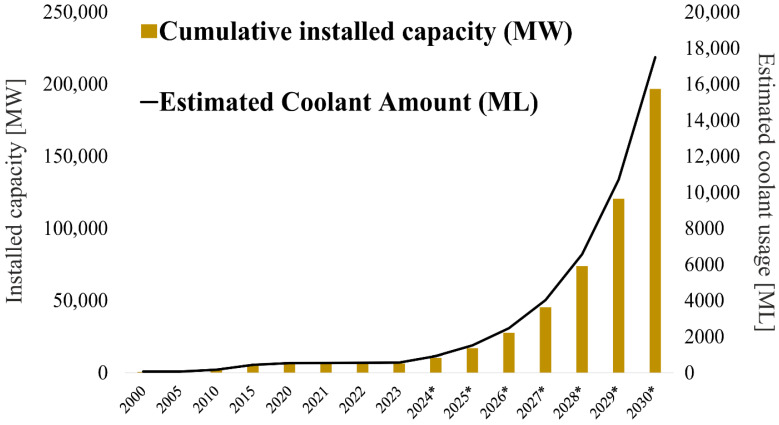
Data on cumulated installed capacity of concentrated solar power (MW) and estimated coolant volume containing glycols (million liters)—historical data for 2023 and forecast for 2024–2030 (marked with *).

The global solar market is growing rapidly, meaning that the demand for glycol-based coolants is also increasing. For a typical single-family home with 100–150 square meters of living space, a 4–5 kW photovoltaic system usually requires 8–13 panels with 350–450 W of power. According to a refrigerant requirement calculator, such a system requires about 20–40 L of glycol-based coolant [7]. Worldwide, according to the International Renewable Energy Agency report “Global Concentrated Solar Power Capacity”, the concentrated solar power (CSP) sector has seen steady progress and diversification since 1990; Global CSP capacity has grown significantly over the past decade, increasing almost fivefold from 1.2 GW in 2010 to around 6.4 GW by 2020. In accordance with the Paris Agreement, it is predicted that the global installed capacity of CSP will require a significant increase, reaching 196.7 GW by 2030 and further expansion to 872.6 GW by 2050. The compound annual growth rate (CAGR) of the cumulative installed capacity for 2024–2030 is approx. 63.12%, which was calculated based on current data and the planned 2030 result of the Paris Agreement. Figure 2 shows the cumulative installed capacity of CSP systems and the estimated volume of coolants required to ensure that these systems operate properly. The graph covers historical data from 2000 to 2023 and a forecast up to 2030. To calculate the amount of fluid required for these systems to function properly, the standard coolant volume requirements per unit of installed power were used. According to the forecast, installed capacity will reach 196,700 MW in 2030, which will require around 17.5 billion liters of glycol-containing coolants.

Combining the projected coolant demand across geothermal and CSP sectors provides a first-order estimate of the glycol waste stream. Given manufacturer-recommended replacement intervals of 5–7 years for closed-loop glycol fluids [8], the ~1600 ML of coolant deployed in geothermal installations by 2030 corresponds to an annual replacement flux of approximately 230–320 ML/year globally, of which 40–50% (92–160 ML/year) is attributable to the glycol fraction alone (typical 40–50% *v*/*v* formulations). The CSP sector adds a substantially larger burden: 17.5 billion liters of deployed coolant by 2030 translates into an annual waste flux of 2.5–3.5 billion liters under the same replacement schedule. Sector-specific data corroborate this scale. Airport de-icing operations alone discharge an estimated 25 million gallons (≈95 ML) of glycol-containing fluids annually in the United States [9]. In the automotive sector, Ben Tarief (2025) [10] reported that >70% of spent coolants in Jordan enter sewage or soil directly, with the national waste stream estimated at several thousand tons per year. Aggregated across renewable energy, transport, and HVAC (heating, ventilation, and air conditioning) sectors, the global flux of spent glycol fluids conservatively exceeds 10^9^ L/year and is projected to grow at 5–6% CAGR through 2030, consistent with the expansion of glycol production markets [11,12].

The growing volumes of waste glycol from renewable energy installations pose environmental risks and require sustainable disposal solutions. Following the criteria of sustainability that will revolutionize world markets in the near future, it is necessary to develop modern, eco-friendly disposal methods that follow the principles of circular economy. The changes in global waste management trends that involve microorganisms for bio-recycling represent a modern approach for combating environmental pollution. Taking into consideration variables such as the growing demand for high-performance vehicles and the need for high-quality components, the demand for coolants will continue to grow. The unquestionable need to maintain the growth rates of the automotive and photovoltaic markets necessitates of regular servicing, which will influence the amount of coolant waste generated every year. This article aims to examine the potential of microorganisms for application in the management of this hazardous waste and to compare these approaches with existing conventional disposal methods.

## 2. Methodology

**Figure 3:**
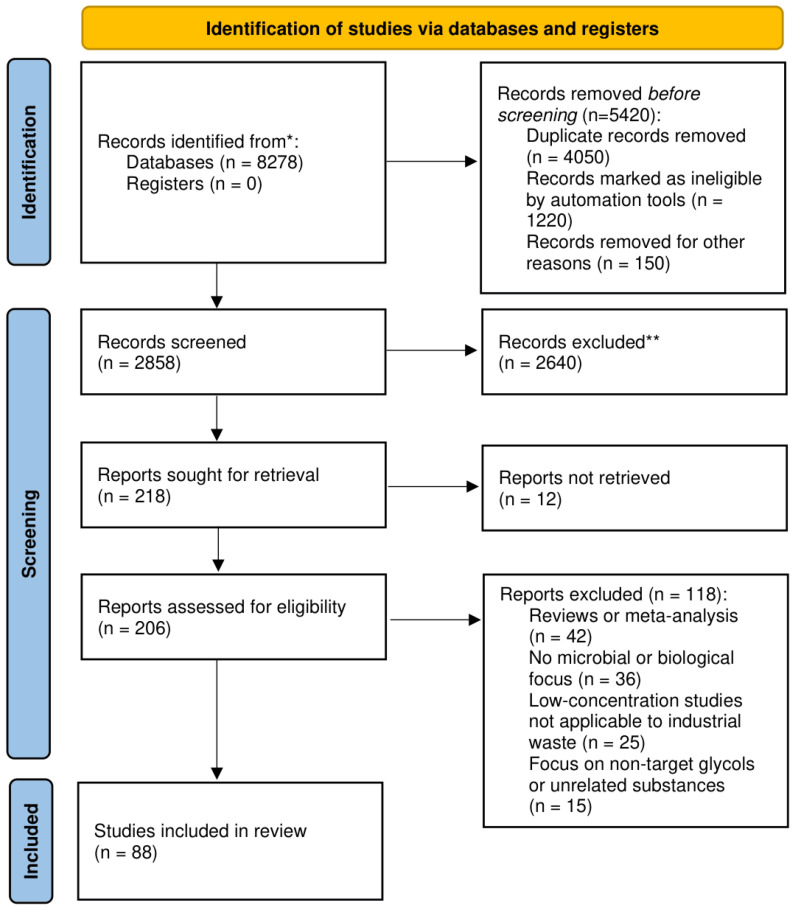
PRISMA flow diagram of literature selection for studies on microbial transformation of glycol waste. * Records were identified from the following databases: PubMed (1328) + Scholar (6950). If database-specific counts were unavailable, the total number of records identified across all databases is reported. ** Records excluded during screening were divided into records excluded by automation tools and records excluded by manual assessment. Automation-based exclusion involved keyword-based filtering of clearly off-topic fields, whereas manual exclusion was based on relevance to the metabolic or biochemical scope of the review.

To ensure a comprehensive and systematic review of the literature on glycol waste and its microbial transformation, we followed PRISMA 2020 (Preferred Reporting Items for Systematic Reviews and Meta-Analyses) guidelines for a transparent and reproducible selection process (Figure 3). Searches were conducted across the academic literature, bioinformatics, and patent databases covering the biodegradation and metabolic pathways of ethylene glycol (EG) and propylene glycol (PG). PubMed, Scopus, and Google Scholar were queried using two complementary search strings: (“ethylene glycol” OR “propylene glycol” OR “glycol-based”) AND (“biodegradation” OR “microbial” OR “biotransformation” OR “bioremediation”) for metabolic pathways, and (“glycol” OR “antifreeze” OR “coolant”) AND (“waste management” OR “recycling” OR “disposal”) AND (“microbial” OR “bacteria” OR “fungi”) for waste management. No restrictive temporal filter was applied, in order to retain foundational studies on glycol catabolism published between 1978 and 2000, which remain the primary source of enzymatic and pathway-level evidence. A supplementary search restricted to 2022 onward was performed in Google Scholar to capture the most recent developments in metabolic engineering, adaptive laboratory evolution, and bioreactor applications. Bioinformatics resources (KEGG, BRENDA, UniProt) were consulted to verify enzymatic pathways and Enzyme Commission (EC) annotations for glycol-degrading enzymes, and patent databases (Espacenet, Google Patents) were queried using the same keyword combinations. Inclusion required peer-reviewed original research or patents addressing microbial degradation or biotransformation of EG or PG; studies were excluded if they were reviews or meta-analyses, lacked a microbial or biological focus, concerned low-concentration conditions not applicable to industrial waste streams, or addressed non-target glycols or unrelated substances.

The initial identification phase yielded 8278 records, a count inflated by the inclusion of Google Scholar, which also contributed to a 48.9% duplication rate upon cross-referencing with structured databases. Before screening, 5420 records were removed: 4050 duplicates, 1220 records flagged as ineligible by automation tools (predominantly citation-only entries lacking full text, market announcements, and non-peer-reviewed patent abstracts), and 150 records excluded for other reasons. Of the remaining 2858 records screened, 2640 were excluded as thematically irrelevant. Of 218 reports sought for retrieval, 12 could not be obtained, leaving 206 for full-text eligibility assessment. A further 118 were excluded at this stage: 42 reviews or meta-analyses, 36 lacking microbial or biological focus, 25 low-concentration studies not applicable to industrial waste, and 15 on non-target glycols or unrelated substances. A total of 88 studies were included in the systematic review.

## 3. Properties and Applications of Coolants

### 3.1. Characteristic of Coolants

Renewable energy systems use heat transfer fluids that contain glycol, water, glycerol, and corrosion and stabilizing additives. These fluids must have the right physical and chemical properties, such as a low freezing point, high boiling point and good thermal conductivity, to ensure that the heating and cooling systems work efficiently. The glycols used in these coolants, propylene glycol (PG) and ethylene glycol (EG), are organic compounds of the diols group.

Propylene glycol (PG), commonly known as 1,2-propanediol, is an aliphatic, hygroscopic alcohol that possesses one primary and one secondary hydroxyl group. It is a stable, viscous, and colorless liquid with a slight odor [13]. PG has a low melting point (−59 °C) and a high boiling point (188.2 °C). This compound is highly miscible with water and many organic solvents, which makes it versatile for a variety of applications [14]; however, it is not found in nature. It is most often is produced by the hydrolysis of propylene oxide with water using the hydrochloric acid process and the peroxidation process [15]. PG is generally recognized as safe by the US Food and Drug Administration (FDA) and is approved for use in food and pharmaceuticals [16]. It has low acute oral toxicity, with an LD50 (median lethal dose) of 20 g/kg in rats, indicating that it is relatively safe for consumption and use in a variety of products [15]. PG is quickly absorbed and processed by the body, with a half-life of about 4 h in adults; nevertheless, its elimination half-life may be longer in infants. Its toxicity is rare and usually occurs under unusual conditions rather than with regular use. It can cause metabolic problems and affect the central nervous system. Especially high doses, as in some medications or treatments, can lead to toxicity. Following absorption, propylene glycol is metabolized by the liver to pyruvic acid and lactic acid or excreted by the kidneys as propylene glycol or as a glucuronide conjugate [13,17].

EG is the simplest sugar alcohol and polyol, which is colorless, odorless, and of relatively high density [18]. Ethylene glycol itself is relatively non-toxic, but its metabolites are highly toxic. The main poisonous metabolite is glycolic acid, which can lead to life-threatening metabolic acidosis. Other metabolites include glyoxylic acid and oxalic acid, with oxalic acid forming insoluble calcium oxalate crystals that can cause tissue damage, particularly to the kidneys [19]. EG is quickly and completely absorbed by the gastrointestinal tract after oral intake. Maximum concentrations are reached within 1–2 h after ingestion. Its metabolism is carried out by enzymes such as alcohol dehydrogenase and aldehyde dehydrogenase, analogous to the metabolism of ethanol [20,21,22]. Ethylene glycol levels above 20 mg/dL are considered toxic and may require therapeutic intervention with ethanol or fomepizole. Levels above 50 mg/dL are critical and may require hemodialysis [23]. Ingesting it in large quantities can lead to central nervous system (CNS) toxicity similar to that caused by ethanol. Toxic metabolites induce severe metabolic acidosis and hypocalcemia, resulting in CNS depression, cardiopulmonary failure, seizures and coma, leading to a deterioration of CNS function [24,25].

### 3.2. Industrial Applications and Consumption Trends

Both propylene (PG) and ethylene (EG) glycol are widely used in industry. Currently, PG is used, for example, as a working fluid in hydraulic presses, a coolant in cooling systems, a cryopreservation of animal organisms, and a preservative and/or emulsifier in food production [26]. Today, PG serves as a necessary building block for making solvents and plasticizers within the chemical sector. Paint and varnish manufacturers select propylene glycol due to its low toxicity level which ensures product users remain protected. Polyester resin manufacturing utilizes propylene glycol as a plasticizer, thus producing marine materials and synthetic marble with sufficient flexibility. The products acquire increased durability and withstand many environmental conditions because of this feature. Flight operations heavily depend on propylene glycol as they incorporate it into anti-freeze and de-icing solutions for their planes, since ethylene glycol (EG) poses higher toxicity levels. Listed as additive E1520, this compound serves multiple purposes in food production, cosmetics, and animal feed. The compound enhances stability and moisturizing properties that have established it as an essential component in everyday consumer products. The pharmaceutical industry benefits from propylene glycol because the substance boosts preparation stability and functions as an excipient for various active pharmaceutical ingredients that enhance their efficacy. Propylene glycol functions as an ideal lubricant and solvent in textile production since it facilitates the processing of dyes through fabrics [13,27,28,29,30].

EG is widely used as an industrial chemical, especially as the main ingredient in antifreeze and de-icing solutions due to its high boiling point (197 °C) and low freezing point. It is also used in brake fluids and other industrial applications as an energy carrier in heat pumps and photovoltaics, and the production of polymers [19].

Ethylene glycol is widely used (approximately 50% of global production) in the automotive industry as an engine coolant, where it is typically used in the form of an aqueous solution. It also performs a heat transfer function in closed heating/cooling systems, air conditioners, and industrial refrigeration units due to its high thermal stability. It usually acts as a precursor to coolants in such installations. It is a key raw material for the production of polyethylene terephthalate (PET) used in plastic bottles, packaging, and fibers. It is an intermediate product in the plasticizer industry, where it is used in the production of alkyd resins, polyurethanes, and plasticizers for paints and adhesives. In the energy sector, it is used to remove water vapor from natural gas pipelines, thus preventing the formation of hydrates. It is the main component of base fluids in some hydraulic systems. It is used to de-ice aircraft and runways, but as previously stated, due to its high toxicity, its use in this regard is limited. It acts as a solvent in industrial paints, printing inks, and wood stains. Due to its high heat capacity, it is used as a coolant in specialized high-performance electronics. In addition to the above, it is a dielectric fluid in some capacitors and a precursor to nitroglycol, dynamite components, and some explosives.

Glycols are also used as heat transfer fluids in HVAC systems and as hydrate inhibitors in the gas industry [31], but the scale of their use raises a serious environmental problem: thousands of tons of ethylene glycol are improperly disposed of each year, and studies conducted in Jordan have shown that over 70% of used coolants from car repair shops go directly into the sewage system or the environment, posing a threat to aquatic ecosystems and public health [32,33]. Comparable patterns are documented in North America and Europe: the U.S. EPA (Environmental Protection Agency) Effluent Limitations Guidelines for Airport Deicing estimate annual aircraft de-icing fluid use at ~21 million gallons of aircraft deicing fluid (ADF), of which 80–90% is PG- or EG-based, with 40–80% entering stormwater or groundwater depending on collection infrastructure [9].

The increasing demand for coolants can be seen very clearly when analyzing the global glycol market. The global market for the production of glycols, according to available reports, was worth $43.8 billion in 2022, and the amount of the compound produced was 30.2 million tons. Predictions for the development of the market point to its dynamic growth. In 2030, the production of glycols (PG and EG) could reach 40.6 million tons [11,12]. The ethylene glycol (EG) production market is estimated to reach $57.5 billion by 2030, with a compound annual growth rate of 5.8% [12]. For propylene glycol (PG), the market size was $4.53 billion in 2021, and the annual growth rate over the next few years (estimated) will be 4.5% [12,34]. The global market related to antifreeze production showed a value of $5.3 billion in 2022 with a growth rate of 1.2%, projected to grow to $5.9 billion by 2030 [35]. Unfortunately, the latest data on Poland’s share of the global market for the production of both individual glycols and refrigerants containing glycols are unavailable. Recent estimates indicate that in 2018, Poland’s annual refrigerant consumption was 76.2 thousand tons, and the value of the legal refrigerant market was 360 million PLN [36].

Analysis of annual global data on glycol consumption demonstrates the high demand for a fast, safe, and efficient method of disposing of liquids containing these substances. In 2007, the annual consumption of ethylene glycol was 18.27 million tons; in 2017 consumption increased to 26.66 million tons, representing more than 45% growth over 10 years [37]. Analysis of available literature data and reports containing predictions for the next several years indicates that demand and production of glycols will expand. Consistently, the amount of waste glycol-based antifreeze generated will also steadily increase. The current noticeable trend in Europe and the world in the waste management sector is the use of methods that are least harmful to the environment. The progressive modernization of this sector in terms of optimizing waste treatment methods, resulting primarily in a significant increase in the percentage of waste recycled, reflects the growing demand for the implementation of new methods to stop or at least slow down the ongoing environmental crisis.

### 3.3. Environmental Impact and Toxicity Concerns

**Figure 4:**
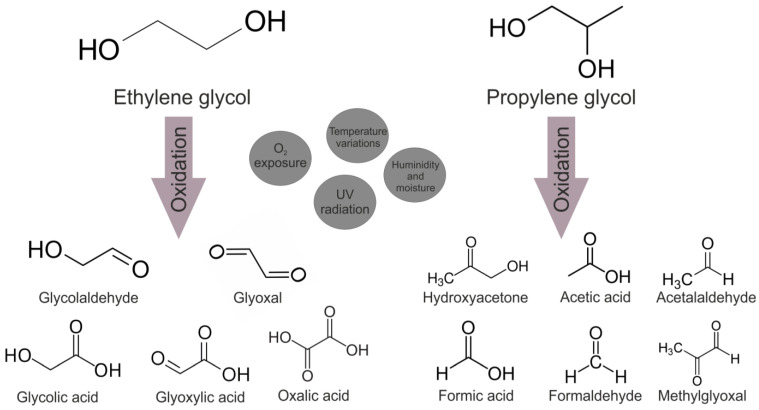
Toxic compounds released during oxidation processes.

Glycols undergo oxidation reactions during heating and air exposure. Therefore, when using carriers composed of superheated glycols, additional components, such as organic acids, aldehydes, and ketones, are to be expected in amounts greater than originally declared by the manufacturer. EG is a compound that is resistant to complete oxidation, but during the aging processes that occur (including partial oxidation), glycolaldehyde and glycolic acid are formed [38]. Among these by-products, glycolic and oxalic acids are the principal drivers of toxicity: in humans and animals, glycolic acid induces severe metabolic acidosis, while calcium oxalate crystals precipitate in renal tubules, causing the acute kidney injury responsible for EG poisoning mortality [10]. The same metabolites underlie the environmental hazard of EG release, since their high biochemical oxygen demand and low-pH character translate directly into the aquatic and soil impacts documented for spent coolant discharge [39]. Toxic compounds released during oxidation are shown in Figure 4.

## 4. Current Waste Management Methods

**Figure 5:**
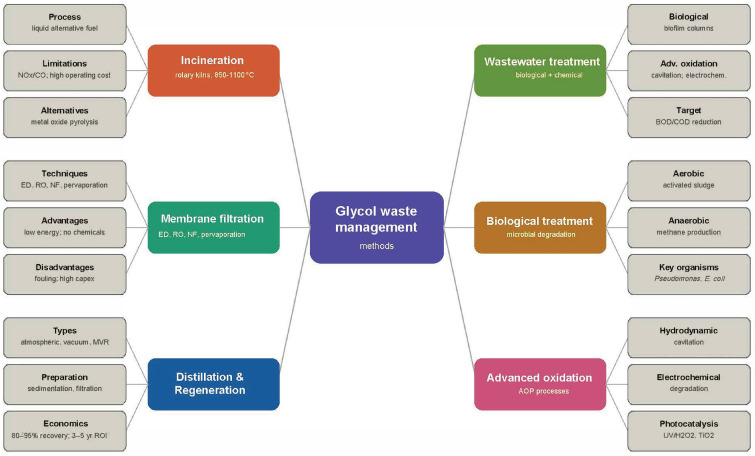
Glycol waste management: conventional treatment methods and biological alternatives.

The current technological solution used in the disposal of thermally degraded superheated cooling liquids is the combustion process in rotary kilns. The minimum temperature required to reduce the volume of this type of waste is 850 °C [40,41]. This method is used in Poland and abroad. Another method, used in the automotive sector, is the process of recovering glycol from used coolants by membrane filtration [42]. The technology used for treating wastewater contaminated with glycol-containing cooling fluids is based on the separation of the solution on a membrane containing concentrated sodium carbonate. The next stage of this technology is a membrane separation process using reverse osmosis, allowing the separation of water and the concentration of the glycol solution. The recovered glycol can be successfully used in an ethylene oxide and glycol production system. However, the developers pointed out that the developed technology has low energy intensity [43]. Short overview of glycol waste management methods is shown in Figure 5.

### 4.1. Incineration Methods

Incineration is one of the methods of utilizing coolants that contain glycols, used mainly when their regeneration is not economically viable or when they are heavily contaminated. The process involves the controlled thermal decomposition of organic compounds at temperatures of 850–1100 °C, ensuring adequate retention times and emission control systems [44]. Ethylene glycol has a combustion heat of approximately 1053 kJ/mol (approximately 17 MJ/kg) [45], which allows it to be used as a fuel. Used solvents, including glycols, are used as liquid alternative fuels in cement kilns as part of the waste co-processing process [46].

However, this method has significant limitations. Firstly, the combustion of glycols generates emissions of toxic compounds, including nitrogen oxides (NO_x_), carbon monoxide (CO), and incomplete combustion products, which require advanced exhaust gas treatment systems [44]. Secondly, it leads to a total loss of the chemical value of glycol, which is contrary to the principles of the circular economy, unlike regeneration methods, which allow for the recovery of a useful product [10]. Thirdly, maintaining combustion facilities involves significant energy inputs and operating costs.

Alternatives to conventional incineration have been investigated, such as metal oxide-catalyzed pyrolysis, which, in the context of PET recycling involving ethylene glycol, can lead to valuable chemical products such as benzene [47]. However, these methods are more suitable for processing plastics than for the direct disposal of glycol fluids.

Nowadays, incineration is treated not as the method of first choice, but as a last resort, for fractions unsuitable for economic regeneration or as an energy source in integrated waste management systems. The priority remains methods of recovery and reuse of glycols, in line with the principles of sustainable development [10].

### 4.2. Membrane Filtration

Membrane filtration is one of the most promising and energy-efficient conventional methods for purifying glycol-containing coolants, offering selective separation of contaminants with significantly lower energy consumption than thermal methods.

Among membrane techniques, electrodialysis (ED), which uses an electric gradient to separate ions, has proven to be particularly effective. Laboratory and pilot-scale studies by Li et al. have demonstrated desalination efficiencies exceeding 90% for ethylene glycol solutions from methane hydrate extraction, at current densities of 10–40 mA/cm^2^ and significantly lower energy consumption than vacuum distillation [48]. Pressure membrane methods, nanofiltration (NF, 5–40 bar) and reverse osmosis (RO, 15–80 bar), are also effective in removing ethylene glycol from wastewater, as confirmed by Nezhad et al. (2022) research on wastewater from the South Pars gas complex [49].

Pervaporation, which combines membrane separation with evaporation at temperatures of 30–80 °C, is also a promising technique. New composite membranes based on graphene oxide-modified polyphenylene oxide show improved water–glycol separation selectivity due to increased hydrophilicity [50]. Pervaporation is particularly attractive as the final purification step in hybrid processes. Dynamic membranes with a polystyrene layer for concentrating contaminants in coolants have also been investigated, although previous work has mainly focused on cutting fluids [51].

Membrane methods offer numerous advantages: low energy consumption, separation selectivity, operation at moderate temperatures, modularity, and no need for chemicals [48,49,50]. Regarding industrial scalability, pilot-scale three-stage electrodialysis treating 40–60 wt% saline EG solutions has demonstrated continuous operation with EG recovery exceeding 99% and energy consumption of 10.4–16.2 kWh/m^3^, at estimated treatment costs below 4 USD per cubic meter of recovered lean-EG, figures substantially lower than those reported for thermal desalination [48]. However, scale-up is constrained by membrane fouling from suspended solids, corrosion inhibitor additives and thermal degradation products present in spent coolants, which accelerate ion-exchange membrane deterioration and increases electrical resistance, raising both energy demand and replacement costs. Additional cost drivers at industrial scale include high capital investment for multi-stack configurations and the need for pre-treatment units to remove particulates prior to membrane separation [49,50]. Despite these challenges, membrane filtration, especially electrodialysis and pervaporation, is a promising alternative to conventional methods, and the development of new materials, such as membranes with nanoparticles, opens up further opportunities to improve the efficiency of regeneration processes [50].

### 4.3. Distillation, Re-Rafination and Regeneration

Distillation remains one of the most widely used and reliable methods for regenerating glycol-based coolants, exploiting the differences in boiling temperatures of the components to separate them. Conventional atmospheric or vacuum distillation allows for the recovery of 80–95% of glycol with a purity that meets standards, with an energy consumption of 2–4 MJ/kg of recovered product [52]. Vacuum distillation is preferred because it lowers the process temperature to 120–150 °C (instead of 197 °C at atmospheric pressure), which reduces the risk of thermal degradation of the glycol and lowers energy consumption [44].

Advanced distillation systems with mechanical vapor recompression (MVR) offer energy savings of up to 60% compared to conventional distillation, achieving propylene glycol purity of over 99.5% [53]. Additional energy savings (30–50%) are provided by thermal coupling technology, which integrates heat streams between distillation columns [53].

The effectiveness of distillation depends on the proper preparation of the raw material, including sedimentation (24–48 h, removal of 60–80% of solid impurities), mechanical filtration (5–50 μm, >95% of particles), and final product conditioning, addition of corrosion inhibitors, pH adjustment, and quality control.

The regeneration of glycol fluids is a comprehensive, integrated approach combining various purification techniques to restore the original properties of used fluids. A multi-stage process involving sedimentation, filtration, distillation, and conditioning allows for the production of a product that meets quality standards, at a production cost of 40–60% of the price of fresh fluid and a return on investment period of 3–5 years [10]. Research on the Jordanian recycling system has demonstrated the feasibility of implementing regeneration on a national scale, requiring collection infrastructure, central processing plants (1000–5000 tons/year), and a quality control system [10].

Regeneration is used not only in the automotive industry, but also in aviation (de-icing fluids with a glycol concentration of 50–100%) [54], and in the gas industry (desalination of solutions from methane hydrate extraction by electrodialysis) [48]. For diluted glycol solutions in wastewater, where conventional regeneration is not economically viable, alternative methods are used: biological treatment in biofilm columns (70–95% efficiency, 12–48 h retention time) [55] and advanced oxidation processes, hydrodynamic cavitation with persulfate [56] or electrochemical degradation with a boron-doped diamond (BDD) anode (mineralization >90%) [33]. However, these methods are used for the complete degradation of glycol, not its recovery.

The regeneration of glycol fluids is the most sustainable approach to managing this waste, in line with the principles of the circular economy, offering significant economic and environmental benefits. The development of energy-efficient technologies (MVR, thermal coupling) and alternative wastewater treatment methods is expanding the possibilities for effective management of various waste streams containing glycols [33,53,55].

### 4.4. Limitations and Environmental Costs

Disposal and regeneration methods for glycol-based coolants can vary significantly in terms of efficiency, cost, and environmental impact. Distillation, despite offering the highest recovery efficiency (80–95%) and product purity comparable to fresh raw material, is characterized by high energy consumption (2–4 MJ/kg) and significant CO_2_ emissions [1,5]. Advanced distillation systems with MVR thermal pumps reduce energy consumption by approximately 60% but require high capital investment [5]. Membrane methods (electrodialysis, pervaporation, nanofiltration) are more energy-efficient and generate lower emissions, but they have limited selectivity, they only effectively remove certain types of contaminants, and also involve costly membrane replacements and the need for integration with other techniques to achieve the required product purity [4,7,22]. Multi-stage regeneration combining sedimentation, filtration, and distillation can handle the widest range of contaminants, but it is a time-consuming process (sedimentation requires 24–48 h) and generates numerous waste streams that require further treatment [1,8]. All of the methods mentioned, both thermal and membrane, generate secondary waste (bottom sediments, concentrates), the disposal of which places an additional burden on the environment [8,22]. At the same time, the problem of improper management of used glycol fluids remains serious: studies indicate that over 70% of used fluids are discharged directly into the environment, which, given the high biochemical oxygen demand of ethylene glycol, leads to eutrophication of water bodies [3,15].

The limitations of existing physicochemical and thermal methods such as high energy consumption, generation of secondary waste, operating costs, and incomplete ability to eliminate all types of pollutants indicate the need to seek alternative solutions, including biological methods, that could enable more sustainable and energy-efficient degradation or regeneration of glycol fluids.

## 5. Microbial Degradation of Glycols

Research to date showed that the concentration of EG, at which microbes can adapt is 10%; therefore, biodegradation in the soil will not proceed evenly [57] and as a result, the toxic compound can contaminate groundwater. It is worth noting that glycol-based fluids in modern heating systems are more concentrated than those tested.

The soil microorganisms used in the biodegradation study of EG [12] demonstrated the ability to take up and assimilate ethylene glycol. These microorganisms include *Pseudomonas aeruginosa*, *Pseudomonas putida*, *Rhodotorula rubra*, *Aspergillus* sp., *Agrobacterium radiobacter*, and *Arthrobacter* sp. [1,58,59,60]. The EG concentrations used in these studies were significantly lower than the actual concentrations found in the coolants.

### 5.1. Bacteria

Bacteria constitute the best-studied group of microorganisms capable of metabolizing glycols, employing diverse strategies depending on oxygen availability. Under aerobic conditions, strains of the genera *Pseudomonas* and *Paracoccus* play a key role, whereas in anaerobic environments, processes based on the dehydration of diols in specialized bacterial microcompartments (BMCs), characteristic of acetogens and certain pathogens, dominate.

The model organism *Pseudomonas putida* KT2440 possesses the genetic potential to degrade ethylene glycol (EG), but its natural efficiency is limited by strict transcriptional regulation. Adaptive Laboratory Evolution (ALE) studies have shown that in *Pseudomonas putida* KT2440 the key regulatory barrier is the GclR repressor, which blocks expression of the *gcl* operon encoding glyoxylate carboligase and downstream enzymes required for glyoxylate assimilation. Mutations in the regulatory gene gclR or engineered overexpression of the gcl operon allow the pathway to be unblocked and enable efficient growth on EG as the sole carbon source [61,62]. In this pathway, EG is sequentially oxidized to glycolaldehyde, glycolic acid, and glyoxylate, which then enters the central metabolism [59]. Moreover, the EG assimilation can be improved by the heterologous expression of the β-hydroxyaspartate cycle from *E. coli*, which allows for a more efficient route for C2 assimilation [63]. In contrast, *Paracoccus* denitrificans bacteria exhibit a naturally high capacity for EG assimilation due to efficient NAD-dependent dehydrogenases, which allow them to accumulate polyhydroxybutyrate (PHB)—a biodegradable bioplastic—directly from monomers recovered from PET waste [64].

Under anaerobic conditions, the degradation mechanism is fundamentally different and relies on the activity of diol dehydratases (PduCDE). The acetogenic bacterium *Acetobacterium woodii* metabolizes EG by dehydrating it to acetaldehyde, which is then disproportionated to ethanol and acetyl-CoA [65]. This process is coupled with the Wood–Ljungdahl pathway and occurs within bacterial microcompartments (BMCs), which protect the cell from aldehyde toxicity. A similar mechanism is used for the degradation of propylene glycol (1,2-propanediol) by bacteria such as *Listeria monocytogenes* and *Salmonella*. In their case, the *pdu* gene cluster encodes enzymes that convert PG to propionate and propanol, which constitute a significant energy source in anaerobic ecological niches [66].

Bacteria are becoming a key component of modern biorefinery strategies. Engineered *Escherichia coli* strains have been successfully programmed to convert ethylene glycol (derived from the hydrolysis of PET bottles) into high-value compounds such as L-tyrosine or glycolic acid [67]. Genetic engineering tools have also been used to improve the EG uptake and cell growth through overexpression of two native genes, alcohol oxidoreductase (*FucO*) and aldehyde dehydrogenase (*AldA*), which are under strict regulatory control, thereby limiting the natural capability to assimilate EG [68]. The native genes were also replaced by heterologous expression of genes encoding enzymes with similar functions, such as *Gox0313* (replacing native *FucO*), to better regulate the pathway of EG assimilation and shift the carbon flux towards glycolic acid [69]. Additional modifications, such as overexpression of *purH* and *metF*, can further increase the yield of glycolic acid production from EG [70]. The use of synthetic bacterial consortia, which share metabolic tasks, allows for overcoming the toxicity of intermediates and the efficient processing of crude plastic waste hydrolysates. It should be noted, however, that while bacterial systems show great potential in converting diluted recycled monomers, their direct application to the treatment of concentrated refrigerant fluids may be limited by high sensitivity to osmotic stress, making the optimization of these strains a key challenge for the future.

### 5.2. Fungi

Filamentous fungi, due to their morphology and ability to synthesize a broad spectrum of extracellular enzymes, play a key role in the treatment of wastewater with a complex chemical matrix. Their application is particularly effective in bioremediation processes, where the goal is the mineralization of pollutants and the reduction in organic load. A strain of *Aspergillus tubingensis* isolated from gas field wastewater demonstrated a significant ability to degrade EG reducing its concentration by over 40% within 240 h. This process was accompanied by a simultaneous 65% reduction in chemical oxygen demand (COD), indicating effective mineralization of organic pollutants in a challenging industrial environment [71].

The adaptive capabilities of filamentous fungi are further supported by studies on *Pseudocochliobolus verruculosus*. It has been demonstrated that the presence of ethylene glycol does not inhibit the growth of this ascomycete; on the contrary, it stimulates the secretion of ligninolytic enzymes, such as laccases and lignin peroxidases [72]. This phenomenon paves the way for the design of processes for the simultaneous removal of glycols and other recalcitrant pollutants, such as industrial dyes.

In the context of propylene glycol (PG), knowledge regarding its mycoremediation remains fragmentary; however, recent studies on plastic degradation shed new light on the sources of this compound in waste. PG is an intermediate product of the biodegradation of polyurethanes (PUR) by fungi of the genus *Fusarium*, including *Fusarium vanettenii* [73]. PG is released as a result of the hydrolysis of the polymer’s ester bonds, catalyzed by specific extracellular lipases (FvLIP1, FvLIP2) and cutinases. Despite confirmation that PG acts as an intermediate metabolite, the mechanism of its further mineralization by fungi remains poorly understood and represents a significant research gap requiring further analysis [73].

### 5.3. Yeast

Unlike bacteria, yeast—and in particular oleaginous species—exhibit a distinctive capacity to thrive in environments with high osmotic pressure, making them ideal candidates for processing concentrated coolants. The recent literature reports indicate a paradigm shift in the approach to these microorganisms: from simple biodegradation toward the biotransformation of glycols into value-added products. Particular attention has been paid to the yeast *Rhodotorula toruloides*, which has been identified as a promising biocatalyst in a circular economy. These strains possess a metabolic pathway enabling the conversion of ethylene glycol (EG) into glycolic acid (GA) with a yield reaching 100% mol/mol. This process is co-metabolic and occurs most efficiently in the presence of a carbon co-substrate, such as glucose, with pathway initiation occurring under nitrogen-limiting conditions [74].

An equally important species is *Yarrowia lipolytica*, known for its robustness in industrial processes. This yeast is characterized by exceptional tolerance to osmotic stress, maintaining metabolic activity at EG concentrations reaching up to 2 M (approx. 12%), which corresponds to the conditions prevailing in partially diluted refrigeration waste. EG conversion by *Y. lipolytica* is a strictly aerobic process. Increasing the mixing speed in the culture medium (from 350 to 450 rpm) leads to a 1.12-fold increase in glycolic acid production, confirming the potential of this yeast in aerobic waste valorization processes [75].

### 5.4. Pathways

Understanding the mechanism of assimilation of glycols contained in refrigeration fluids is the first step toward developing a method for their bio-recycling. Optimizing the cultivation process in terms of efficient growth of microorganisms on waste glycols will make it possible to obtain biomass that can be an immunostimulatory animal feed or feed supplement. The idea of obtaining high-energy animal feed through glycol biotransformation would certainly contribute to maintaining the sustainable development of our civilization, especially in light of the recent ecological problems we face [58,60,76]. The use of unmodified microorganisms has the additional benefit of direct environmental application (e.g., in bioremediation) without additional ethical constraints. The glyoxylate, dicarboxylate, and propionate pathways are the primary metabloic routes involved in the conversion of EG and PG. Biodegradation of propylene glycol can occur under aerobic and anaerobic conditions. During the anaerobic decomposition of propylene glycol, propionaldehyde is formed, which is then converted to propionate and 1-propanol. The resulting propionate undergoes further metabolic transformations to acetate, methane, and carbon dioxide [77].

#### 5.4.1. Propylene Glycol

Propylene glycol (1,2-propanediol, 1,2-PD) is broken down primarily by bacteria via several distinct enzymatic pathways, each of which leads, via common intermediates—propionaldehyde and propionyl-CoA—to the final products of fermentation or respiration.

The pathway encoded by the pdu operon, the best-known and most extensively described pathway for the catabolism of 1,2-PD, relies on coenzyme B12 (adenosylcobalamin, Ado-B12) (*pdu*). This pathway begins with the conversion of 1,2-propanediol to propionaldehyde by Ado-B12-dependent diol dehydratase. Propionaldehyde is then catabolized to propionic acid and propanol, likely by coenzyme A (CoA)-dependent aldehyde dehydrogenase, phosphotransacylase, propionate kinase, and alcohol dehydrogenase [78]. The enzymes of the pathway are encoded in the pdu operon. The catalytic process involves PduCDE (coenzyme B12-dependent diol dehydratase), PduP (propionaldehyde dehydrogenase), PduL (phosphotransacylase), and PduW (propionate kinase), leading to propionate, or alternatively to 1-propanol via PduQ (alcohol dehydrogenase) [79]. Bacterial microcompartments (BMCs) are a critical part of the pathway. The first two steps of 1,2-PD degradation occur in the lumen of the Pdu MCP (microcompartment), where 1,2-PD is converted to propionaldehyde and then to propionyl-CoA by B12-dependent diol dehydratase (PduCDE) and propionaldehyde dehydrogenase (PduP). Propionyl-CoA then leaves the MCP into the cytoplasm, where it is converted to propionate or enters the central metabolism via the methylcitrate pathway [80]. The protective role of microcompartments has been well documented experimentally. The major products of the aerobic degradation of 1,2-PD are propionaldehyde, propionate, and 1-propanol. A mutant strain lacking functional MCP accumulated propionaldehyde at a concentration 10 times higher than that of the wild-type strain (15.7 mM versus 1.6 mM), indicating a link between this compound and growth inhibition [81].

An alternative, cobalamin-independent pathway for 1,2-PD catabolism on glycyl radical-containing enzymes. The *grp* (*glycyl radical propanediol*) gene cluster identified in *E. coli* CFT073 contains 21 genes transcribed in the same direction. Sequence analyses indicate that the *grp* genes encode enzymes involved in the metabolism of 1,2-PD to propionate and 1-propanol (including GR-DDH—glycyl radical diol dehydratase), five MCP envelope proteins, and a number of proteins of unknown function [82]. The reaction mechanism of GRE (Glycyl Radical Enzyme) differs fundamentally from that of the B12-dependent enzyme. Propane-1,2-diol dehydratase, a member of the GRE family, utilizes protein-based radicals to catalyze the chemically demanding dehydration of (S)-1,2-propanediol. According to the results of ^18^O isotope labeling, GRE and B12-dependent dehydratase employ distinct mechanisms—GRE appears to catalyze the direct elimination of a hydroxyl group from the initially formed substrate radical, bypassing the generation of a 1,1-geminaldiol intermediate [83]. The GRE pathway is biologically significant for bacterial pathogenicity. Tests conducted on the *E. coli* reference collection (ECOR) showed that more than 10% of *E. coli* strains ferment 1,2-PD using a glycyl radical microcompartment [82]. The enzymatic organization within the GRM (Glycyl Radical Microcompartment) is analogous to the pdu pathway. In the general model of metabolosome function, signature enzymes participate in the formation of an aldehyde, which is then oxidized to acyl-CoA thioester by acyl-aldehyde dehydrogenase (AldDH) or reduced to an alcohol by alcohol dehydrogenase (ADH). Acyl-CoA is further converted to acyl phosphate by phosphotransacylase (PTA), and then to free carboxylic acid by kinase (AcK) with the production of ATP [84].

Several types of bacteria, including *Salmonella*, *Klebsiella*, *Shigella*, *Yersinia*, *Listeria*, *Lactobacillus*, and *Lactococcus*, contain species capable of growing on 1,2-propanediol in a manner dependent on coenzyme B12. The compound 1,2-PD is the major product of the anaerobic degradation of rhamnose and fucose—common sugars found in plant cell walls, bacterial exopolysaccharides, and intestinal epithelial glycoconjugates [81].

Both environmental conditions and the availability of electron acceptors determine the final metabolic products. Propionaldehyde is converted to propionate by CoA-dependent propionaldehyde dehydrogenase (PduP), phosphotransacylase (PduL), and propionate kinase (PduW), or to 1-propanol by alcohol dehydrogenase (PduQ). The degradation of 1,2-PD yields ATP and propionyl-CoA, which enters the central metabolism via the methylcitrate pathway [79].

**Figure 6:**
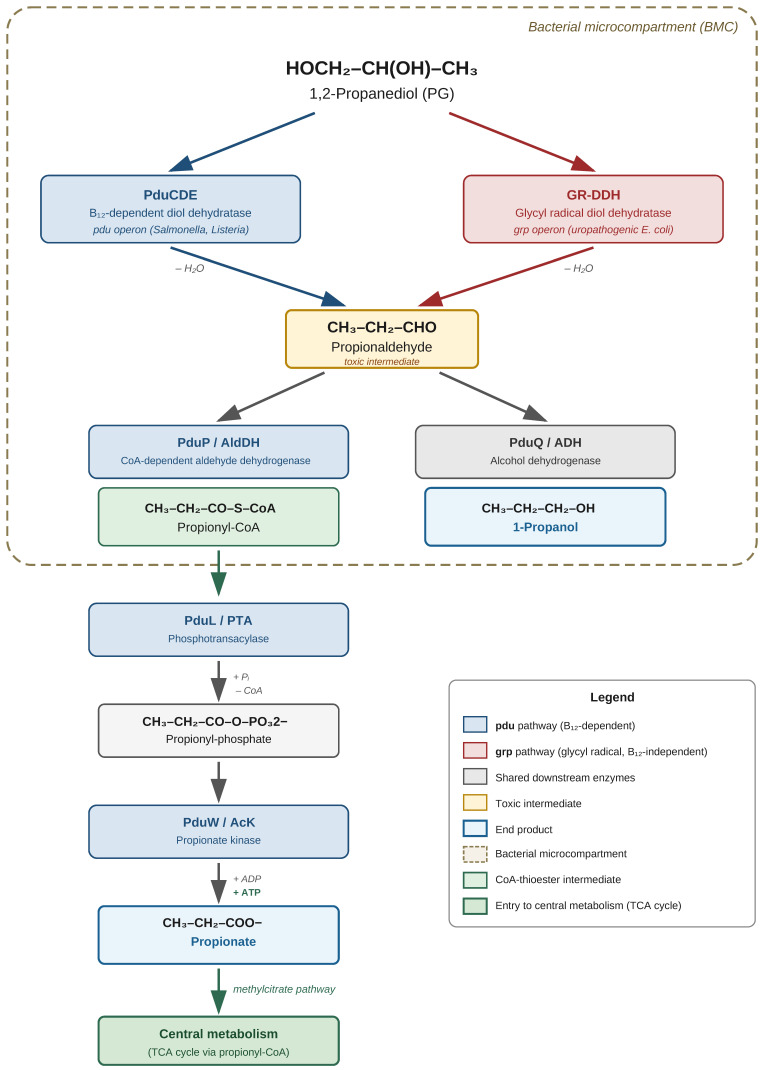
Microbial metabolic pathways for 1,2-propanediol (PG) degradation in bacteria. PG is dehydrated to propionaldehyde via two parallel routes: the B_12_-dependent pathway catalyzed by PduCDE (*pdu* operon; *Salmonella*, *Listeria*, *Klebsiella*) and the cobalamin-independent glycyl radical pathway catalyzed by GR-DDH (*grp* operon; uropathogenic *Escherichia coli*). Propionaldehyde is a toxic intermediate sequestered within bacterial microcompartments (BMCs; dashed boundary) and is further metabolized to propionyl-CoA by CoA-dependent aldehyde dehydrogenase (PduP/AldDH) or reduced to 1-propanol by alcohol dehydrogenase (PduQ/ADH). Propionyl-CoA exits the BMC and is converted via phosphotransacylase (PduL/PTA) and propionate kinase (PduW/AcK) to propionate with concomitant ATP generation; propionate subsequently enters central metabolism through the methylcitrate pathway. Shared downstream enzymes (grey) operate in both *pdu* and *grp* systems.

A schematic representation of the proposed microbial metabolic pathways for ethylene glycol degradation—encompassing the cytoplasmic NAD-dependent oxidative route, the PQQ-dependent periplasmic oxidation, the mycofactocin-dependent pathway, and the anaerobic B12-dependent dehydration route—is shown in Figure 6; the corresponding enzymatic systems, end products, and representative organisms are summarized in Table 1.

#### 5.4.2. Ethylene Glycol

Ethylene glycol (EG) is degraded by microorganisms through two main pathways: aerobic oxidation and anaerobic dehydration. The subsequent fate of the intermediate products depends on the availability of cofactors, oxygen conditions, and the enzyme machinery of the cell.

The oxidative (aerobic) pathway is the most extensively described and best-understood route of EG catabolism, involving sequential oxidation to glyoxylate, which then enters the central metabolic pathway. Spontaneous *E. coli* mutants capable of growing on ethylene glycol as the sole source of carbon and energy were characterized by two main features: increased activity of propanediol oxidoreductase—an enzyme present in the parent strain that also converts ethylene glycol to glycolaldehyde—and constitutive synthesis of highly active glycolaldehyde dehydrogenase, which converts glycolaldehyde to glycolate. Glycolate was metabolized via the glycolate pathway, as confirmed by the induction of glycolate oxidase in cells grown on EG. Glycolaldehyde and glycolate were identified as intermediate metabolites of the pathway [85]. In *E. coli*, EG is oxidized to glycolaldehyde by the NAD^+^-dependent dehydrogenase activity of FucO, and glycolaldehyde is converted to glycolate by AldA. Glycolate can then enter the central metabolic pathway via the oxidative pathway and/or the glyoxylate side pathway [86]. A detailed description of the subsequent stages in *E. coli* involves four sequential assimilation reactions. Ethylene glycol is sequentially oxidized by lactaldehyde reductase (FucO), aldehyde dehydrogenase A (AldA), and glycolate dehydrogenase (GlcDEF) to glycolaldehyde, glycolic acid, and glyoxylic acid. Glyoxylate can then be converted to 2-phosphoglycerate via the glycolate and glyoxylate degradation pathway I, involving four enzymes in sequence: Gcl, Hyi, GlxR, and GlxK. Alternatively, glyoxylate can be converted to malate via the glyoxylate shunt, catalyzed by GlcB (malate synthase) [87].

In *Pseudomonas putida*, on the other hand, EG oxidation is initiated by PQQ-dependent periplasmic enzymes. PQQ-dependent alcohol dehydrogenases—PedE and PedH—contain the cofactor PQQ (pyrroloquinoline quinone) as a prosthetic group in their structure. These periplasmic enzymes play a key role in the degradation of primary alcohols. In addition to these enzymes, an increase in the expression of two NADH-dependent aldehyde dehydrogenases (ALDHs)—PedI and PP_0545—was observed in the presence of ethylene glycol [59]. A comparison of *P. putida* strains reveals differences in their assimilation capabilities. Strain JM37 grew rapidly using ethylene glycol as the sole source of carbon and energy, whereas strain KT2440 showed no growth after 2 days of cultivation under the same conditions. However, biotransformation experiments demonstrated EG metabolism by both strains, with temporary accumulation of glycolic acid and glyoxylic acid by KT2440. In *P. putida* JM37, in the presence of ethylene glycol or glyoxylic acid, tartrate-semialdehyde synthase (Gcl), malate synthase (GlcB), and isocitrate lyase (AceA) were induced. Under the same conditions, strain KT2440 showed induction of AceA only [59].

Under anaerobic conditions, acetogens employ a different mechanism based on dehydration, analogous to the 1,2-propanediol degradation pathway. Since the PduC subunit of the 1,2-propanediol dehydratase PduCDE is produced in *A. woodii* cells growing on ethylene glycol, and since this is the only dehydratase encoded in the genome of this organism, the initial conversion of ethylene glycol is most likely catalyzed by the same enzyme responsible for the initial dehydration of 1,2-PD. The first intermediate in EG degradation following dehydration is acetaldehyde. Extracts from cells growing on EG can either reduce acetaldehyde with the participation of NADH or oxidize it in a CoA-dependent reaction, transferring electrons to NAD^+^. In this way, acetaldehyde undergoes disproportionation to yield equal amounts of ethanol and acetyl-CoA [65]. While many aerobic bacteria utilize ethylene glycol by oxidizing it to glycolaldehyde and glycolate, followed by an oxidase reaction to form glyoxylate, anaerobic bacteria employ an alternative pathway via acetaldehyde, which is produced by the dehydration of EG catalyzed by a diol dehydratase that is highly sensitive to oxygen [65]. The involvement of bacterial microcompartments in the anaerobic pathway in *A. woodii* has been experimentally confirmed. Biochemical data and protein synthesis analysis results support the hypothesis that the PduCDE (propanediol dehydratase) and PduP (CoA-dependent propionaldehyde dehydrogenase), encoded by the pdu gene cluster, also catalyze the dehydration of ethylene glycol to acetaldehyde and its CoA-dependent oxidation to acetyl-CoA. Furthermore, genes encoding bacterial microcompartments as part of the pdu cluster are also expressed during growth on ethylene glycol, which supports a dual function of the Pdu microcompartment system [65].

**Figure 7:**
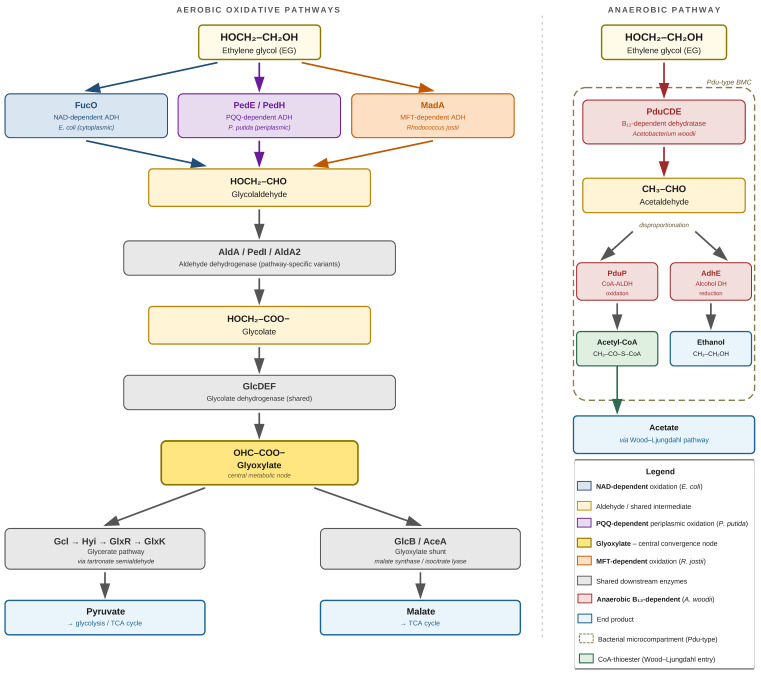
Microbial metabolic pathways for ethylene glycol (EG) degradation under aerobic and anaerobic conditions. Three aerobic oxidative routes initiate the pathway with distinct alcohol dehydrogenases—NAD-dependent FucO in *Escherichia coli* (blue), PQQ-dependent periplasmic PedE/PedH in *Pseudomonas putida* (purple), and mycofactocin (MFT)-dependent MadA in *Rhodococcus jostii* RHA1 (orange)—and converge at glycolaldehyde and glycolate before oxidation to glyoxylate by the shared glycolate dehydrogenase GlcDEF. Glyoxylate, the central metabolic node, is subsequently assimilated either via the glycerate pathway (Gcl-Hyi-GlxR-GlxK, yielding pyruvate for glycolysis/TCA entry) or via the glyoxylate shunt (GlcB/AceA, yielding malate). Under anaerobic conditions (right; red), *Acetobacterium woodii* employs the B_12_-dependent diol dehydratase PduCDE to generate acetaldehyde, which is disproportionated within Pdu-type bacterial microcompartments (dashed boundary) to acetyl-CoA (via CoA-dependent PduP) and ethanol (via AdhE); acetyl-CoA subsequently enters the Wood–Ljungdahl pathway to yield acetate.

Glyoxylate is a key metabolic node for organisms that degrade EG via the oxidative pathway. Two molecules of glyoxylate are condensed to tartronic semialdehyde by glyoxylate carboxylase (Gcl) with the release of one molecule of CO_2_; ultimately, tartronic semialdehyde is metabolized to pyruvate, which can be utilized for growth. Bacteria lacking functional Gcl, such as *P. putida* KT2440, can only oxidize glyoxylate via the glyoxylate shunt, which can only generate energy [74]. Transcriptomic studies have shown that EG catabolism by *Rhodococcus jostii* RHA1 ultimately leads to glycolate, which enters the TCA cycle via a pathway encoded by two Gcl clusters: GCL1 and GCL2. Only GCL2 encodes a GlcD homolog, predicted to oxidize glycol to glyoxylate. The remaining enzymes of the pathway are encoded by both clusters and are predicted to convert glyoxylate to 2-phosphoglycerate, which enters glycolysis [88]. A schematic representation of the proposed microbial metabolic pathways for ethylene glycol degradation—encompassing the cytoplasmic NAD-dependent oxidative route, the PQQ-dependent periplasmic oxidation, the mycofactocin-dependent pathway, and the anaerobic B_12_-dependent dehydration route—is shown in Figure 7; the corresponding enzymatic systems, end products, and representative organisms are summarized in Table 2.

## 6. Challenges

Despite a growing wealth of evidence confirming the ability of microorganisms to metabolize glycols, the practical application of microbial degradation in actual cooling fluid waste streams faces a number of significant and interrelated limitations. These challenges encompass both the physicochemical properties of the substrate itself and the inherent physiological limitations of currently known degrading strains. Three areas are of particular concern: the high glycol concentrations prevalent in industrial heat transfer fluids and their associated osmotic burden; the toxicity of oxidation products that accumulate during the thermal aging of glycol-based fluids; and the sensitivity of microbial metabolism to temperature and pH, both of which deviate substantially from laboratory-optimized conditions in real waste-treatment scenarios.

A pronounced concentration gap exists between laboratory biodegradation studies and real-world applications. Microbial degradation of PG has been investigated at concentrations not exceeding 0.5%, and EG studies rarely surpass 10%, whereas operational heat transfer fluids contain glycol at ≥10%, with antifreeze formulations reaching 100%. Critically, no published study has systematically evaluated the degradation performance of the organisms discussed in Section 5 at glycol concentrations matching those of industrial coolant waste; extrapolation of low-concentration kinetic data to the industrial range is not justified, as elevated osmolarity triggers plasmolysis, inhibits enzymatic activity, and at sufficiently extreme values causes irreversible cell death, while substrate inhibition and aldehyde intermediate accumulation further compromise microbial physiology in a non-linear manner. The chemical complexity of spent coolants compounds the problem. Beyond the glycol itself, these formulations contain corrosion inhibitors, biocides, surfactants, and thermal degradation products that may exert synergistic inhibitory effects independent of osmotic stress. The high COD of concentrated glycol solutions further imposes oxygen limitation in aerobic systems, restricting the availability of the terminal electron acceptor required by most characterized aerobic pathways. Mitigation strategies under investigation—pre-dilution, staged acclimation, fed-batch and membrane bioreactor configurations, and adaptive laboratory evolution—address substrate toxicity only partially; osmotic tolerance remains a complex, polygenic trait not yet systematically engineered in glycol-degrading chassis organisms.

Several strategies are being actively pursued to overcome these barriers. Adaptive laboratory evolution (ALE) has been successfully applied to *Pseudomonas putida* KT2440, yielding variants with unblocked glyoxylate carboxylase expression and substantially improved growth on EG as the sole carbon source [61,62]. Targeted metabolic engineering of the oxidative pathways summarized in Figure 7 and Table 2—particularly overexpression of PQQ- and NAD-dependent dehydrogenases and the downstream Gcl/GlcB glyoxylate assimilation branch—offers a complementary route to increasing flux through rate-limiting steps and preventing accumulation of toxic intermediates such as glycolaldehyde and glyoxylate [67]. Combined with optimization of growth conditions (controlled dilution, staged acclimation, fed-batch feeding), these approaches move glycol-degrading chassis organisms closer to the performance required for industrial waste streams.

Spent glycol-based heat transfer fluids accumulate a spectrum of oxidation and degradation products during prolonged thermal service, whose biological effects on degrading microorganisms are distinct from—and frequently more severe than—those of the parent glycols. In the case of EG, principal oxidation products include glycolaldehyde, glycolic acid, glyoxylic acid, and oxalic acid. Oxalic acid inhibits key TCA cycle enzymes and precipitates calcium ions as insoluble calcium oxalate; glyoxylate, a central intermediate in EG oxidation, accumulates to inhibitory concentrations when downstream enzymatic steps become rate-limiting; glycolaldehyde, as a reactive aldehyde, forms adducts with proteins and nucleic acids, exerting direct cytotoxicity independent of its metabolic role. For PG, the analogous problematic intermediate is propionaldehyde—an obligate product of both the cobalamin-dependent (*pdu*) and glycyl radical (*grp*) pathways. Strains lacking functional bacterial microcompartments (BMCs) accumulate propionaldehyde at concentrations more than ten-fold higher than in wild-type cells, directly correlating with growth inhibition; BMC capacity may be overwhelmed at the high substrate fluxes characteristic of concentrated waste streams. Corrosion inhibitor additives present in commercial formulations introduce a further layer of toxicological complexity: ethanolamine-based inhibitors may act as competitive substrates or inhibitors of glycol-oxidising dehydrogenases, while nitrite-based inhibitors can perturb the redox balance of anaerobic degraders. The net result is that the biological treatability of aged, additive-containing coolant waste is substantially lower than predictions based on studies with analytically pure glycol solutions.

Temperature and pH present practical challenges for glycol biodegradation at operational scale. Most characterized glycol-degrading bacteria exhibit optimal growth in the mesophilic range of 25–37 °C, with sharp activity declines below 15 °C and above ~42 °C, yet glycol waste from renewable energy installations may be collected and processed across the full range of ambient temperatures. In temperate climates, seasonal fluctuations span from sub-zero conditions to summer maxima exceeding 30–a range current strains are ill-equipped to handle without thermal supplementation. Psychrotolerant glycol-degrading microorganisms remain poorly described, constituting a meaningful gap in the field. Temperature also directly affects key catabolic enzymes: cobalamin-dependent diol dehydratases (PduCDE) suffer both thermal inactivation at high temperatures and reduced catalytic efficiency at low temperatures, while glycyl radical enzymes (GREs) of the *grp* pathway require strictly anaerobic conditions to maintain radical cofactor activity, conditions that are non-trivial to sustain at industrial scale alongside narrow temperature control.

pH management is equally complex. Glycol catabolism generates organic acids as obligate intermediates—glycolic acid, glyoxylic acid, and oxalic acid from EG; propionic acid from PG fermentation—and progressive medium acidification suppresses growth and enzyme activity in strains with neutral-to-mildly alkaline optima. Spent coolant fluids compound this by arriving with highly variable initial pH profiles, reflecting the degradation of amine-based inhibitors and accumulated oxidation products that differ between batches depending on service history. Continuous alkali dosing can correct pH but adds operational cost and complexity. Together, these constraints illustrate that bridging the gap between laboratory performance and practical waste treatment remains a substantial engineering and microbiological challenge.

**Figure 8:**
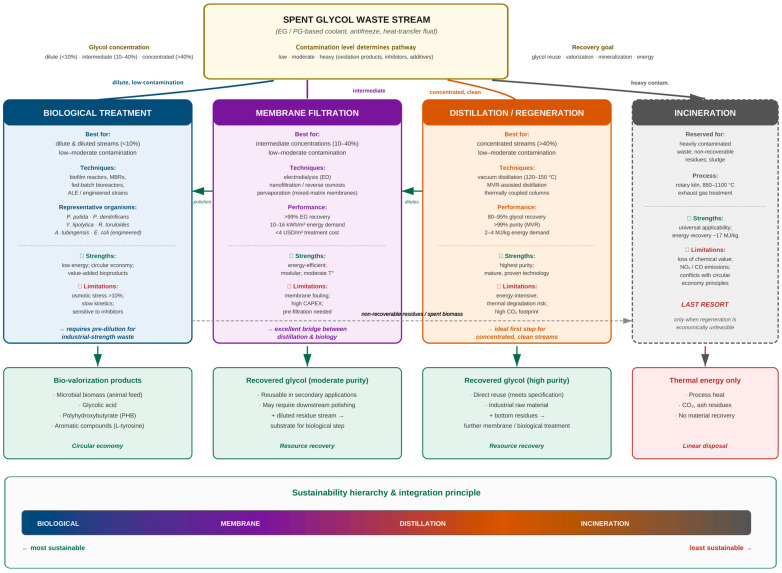
Integrated framework for glycol waste management. Method selection is organized into three tiers: (**top**) characterization of the spent glycol waste stream according to glycol concentration, contamination level, and recovery goal; (**middle**) four processing pathways—biological treatment, membrane filtration, distillation/regeneration, and incineration—each characterized by their applicability window, representative techniques, performance data, strengths, and limitations; (**bottom**) resulting output streams and a sustainability hierarchy indicating the relative environmental favorability of each method. Biological treatment (blue) is best suited for dilute streams (<10%), membrane filtration (purple) for intermediate concentrations (10–40%), distillation/regeneration (orange) for concentrated streams (>40%), and incineration (grey, dashed) for heavily contaminated, non-recoverable residues. Green left-pointing arrows indicate optional integration routes to an earlier treatment step, for example for stream polishing or dilution before further processing.

Despite these constraints, the biological approach does not compete with existing physicochemical technologies but rather complements them within an integrated waste management framework. The selection criteria between methods—glycol concentration, contamination profile, and the targeted processing outcome—and the complementary positioning of biological treatment, membrane filtration, distillation/regeneration, and incineration are summarized in Figure 8. This integration principle positions biological treatment as the most sustainable endpoint of a hybrid processing train in which physicochemical methods serve as pre-concentration and polishing stages, while incineration remains reserved strictly for non-recoverable residues.

## 7. Current and Future Research Directions

Current innovations point toward hybrid and multi-technology approaches that may complement biological treatment at the high concentrations characteristic of industrial waste. Pervaporation-assisted distillation can reduce total annual cost and energy consumption by approximately 25% and 41%, respectively compared to conventional distillation, while advanced mixed-matrix membranes—including polyphenylene oxide/graphene oxide composites and functionalized polymers of intrinsic microporosity—have been developed specifically for high-concentration EG dehydration (70–95 wt%) [50]. Catalytic routes originally developed for PET depolymerization further suggest chemical valorization of EG-containing streams into higher-value products rather than mere recovery [47]. In parallel, adaptive laboratory evolution and targeted genetic engineering of glycol-degrading strains—exemplified by engineered *Pseudomonas putida* KT2440 variants and *Escherichia coli* strains optimized for PET-derived EG utilization—represent the most actively developed biotechnological route to overcoming native concentration and toxicity limits, and may ultimately be integrated with physicochemical pre-concentration in hybrid waste treatment trains [61,62].

Based on the current state of the field, we identify five concrete research priorities that should guide future work on microbial glycol waste management:

(1) Isolation and characterization of psychrotolerant glycol-degrading strains active within the temperature ranges encountered in field applications (seasonal variation from sub-zero to >30 °C), addressing a meaningful gap in the currently mesophilic-dominated literature.

(2) Engineering of osmotic tolerance in established model organisms—through adaptive laboratory evolution (ALE), directed mutagenesis, or heterologous expression of compatible solute biosynthesis pathways—as a prerequisite for processing undiluted or minimally diluted industrial waste streams.

(3) Expansion of bacterial microcompartment (BMC) capacity in PG-degrading strains to prevent propionaldehyde accumulation at high substrate fluxes, requiring dedicated metabolic engineering efforts targeting the *pdu*/*grp* shell protein stoichiometry.

(4) Development of bioreactor configurations (membrane bioreactors, fed-batch systems) that decouple substrate exposure from cell density, enabling scale-up of biodegradation performance from laboratory to operational conditions.

(5) Transition from pure substrates to representative spent coolant matrices containing corrosion inhibitors, biocides, and thermal degradation products, in order to obtain practically relevant performance data. In parallel, comparative life-cycle assessments (LCA) quantifying the carbon footprint of biological treatment relative to incineration and membrane-based regeneration will be essential to support the rational selection of waste management strategies at operational scale.

## 8. Conclusions

Glycol-based coolants generated by the expansion of renewable energy infrastructure present a new waste management challenge that is becoming increasingly significant from both regulatory and environmental perspectives. The growing volumes of spent glycol-based coolants generated by renewable energy installations pose an environmental challenge for which conventional physicochemical methods—incineration and membrane filtration—are energetically and economically unfeasible at the anticipated scale. Catabolic pathways for both EG and PG have now been characterized at the enzymatic level in various bacterial and fungal taxa, and recent work in metabolic engineering demonstrates the potential to increase degradation efficiency and redirect glycol-derived carbon toward value-added products. Nevertheless, osmotic stress induced by industrial glycol concentrations, the accumulation of cytotoxic intermediate products, and the inhibitory chemical matrix of spent coolant formulations collectively limit biodegradation efficiency to levels significantly below those achievable with analytically pure substrates. Bridging the gap between the laboratory and practical application will require advances in osmotic tolerance engineering, the systematic isolation of psychrotolerant strains, the optimization of bacterial microcompartment capacity, and the development of bioreactor configurations adapted to variable real-world conditions. Harnessing the full potential of microbial glycol catabolism within a circular economy requires a decisive shift from studies using pure substrates toward matrices of spent coolants with representative compositions, ensuring that future performance data will have direct practical relevance.

## Figures and Tables

**Table 1:** Microbial pathways for 1,2-propanediol metabolism: enzymatic systems, products and representative organisms.

Pathway	Enzymes	End Products	Representative Organisms
B12-dependent (pdu operon)	Adenosylcobalamin-dependent diol dehydratase (PduCDE); Propionaldehyde dehydrogenase (acylating, PduP); Phosphotransacylase (PduL); Propionate kinase (PduW);1-propanol dehydrogenase (PduQ)	Propionate; 1-propanol (via propionyl-CoA intermediate; ratio depends on redox conditions and MCP function)	*Salmonella enterica* serovar Typhimurium; *Klebsiella pneumoniae*; *Listeria monocytogenes*/*L. innocua*; *Lactobacillus diolivorans*
Glycyl radical enzyme pathway (grp operon, B12-independent)	Glycyl radical 1,2-propanediol dehydratase (Grp enzyme); Acylating aldehyde dehydrogenase (AldDH); Phosphotransacylase (PTA);Acyl kinase (AcK-like, propionate-forming); Alcohol dehydrogenase (ADH)	Propionate; 1-propanol (propionaldehyde as toxic intermediate, sequestered in bacterial microcompartments—Grp-type MCP)	Uropathogenic *Escherichia coli* (e.g., CFT073); *Rhodopseudomonas palustris*; Multiple *E. coli* lineages (including ECOR strains)
Non-specific oxidative pathways (non-MCP, NAD-dependent)	NAD-dependent diol/alcohol dehydrogenases; Aldehyde dehydrogenases (e.g., AldA in *E. coli*)	Pyruvate; acetyl-CoA; acetate (non-specific oxidation products; not a canonical 1,2-PD catabolic pathway)	*Pseudomonas* spp.; *Paracoccus* spp.; Methylotrophic bacteria; Adaptive *Escherichia coli* mutants

**Table 2:** Microbial pathways for ethylene glycol metabolism under aerobic and anaerobic conditions.

Pathway	Enzymes	End Products	Representative Organisms
Oxidative pathway (NAD-dependent, cytoplasmic)	NAD-dependent alcohol dehydrogenases (e.g., FucO/AdhP/YqhD; EG → glycolaldehyde); Aldehyde dehydrogenase (AldA; glycolaldehyde → glycolate); Glycolate dehydrogenase (GlcDEF; glycolate → glyoxylate); Glyoxylate carboligase pathway (Gcl, Hyi, GlxR, GlxK) or malate synthase (GlcB)	Glyoxylate → pyruvate (via tartronate-semialdehyde pathway) or malate (via glyoxylate shunt);glycolaldehyde and glycolate as key intermediates	*Escherichia coli* (adapted mutants with constitutive expression of EG oxidation pathway); *Paracoccus denitrificans* (NAD-dependent Etg-like systems)
PQQ-dependent periplasmic oxidation	PQQ-dependent alcohol dehydrogenases PedE (Ca^2+^-dependent)/PedH (Ln^3+^-dependent; EG → glycolaldehyde); Aldehyde dehydrogenases (PedI, PP_0545/AldB; glycolaldehyde → glycolate); Glycolate dehydrogenase (GlcDEF; glycolate → glyoxylate); Glyoxylate assimilation via Gcl or glyoxylate shunt (AceA, GlcB)	Pyruvate (in strains with functional Gcl, e.g., JM37);glycolate and glyoxylate accumulation (e.g., KT2440 lacking full pathway);possible PHA accumulation	*Pseudomonas putida* JM37; *Pseudomonas putida* KT2440; *Pseudomonas umsongensis* GO16
Mycofactocin (MFT)-dependent oxidation	MFT-dependent alcohol dehydrogenase (MadA; EG → glycolaldehyde); Aldehyde dehydrogenase (AldA2; glycolaldehyde → glycolate); Glyoxylate assimilation via GCL1/GCL2 clusters (glyoxylate → 2-phosphoglycerate → central metabolism)	Glycolate → central metabolism (via 2-phosphoglycerate → glycolysis/TCA cycle); CO_2_ released during glyoxylate assimilation	*Rhodococcus jostii* RHA1
Anaerobic dehydration pathway (B12-dependent, Pdu-type MCP)	B12-dependent diol dehydratase (PduCDE; EG → acetaldehyde); CoA-dependent aldehyde dehydrogenase (PduP; acetaldehyde → acetyl-CoA); Alcohol dehydrogenase (AdhE; acetaldehyde ⇌ ethanol); Phosphotransacetylase + acetate kinase (acetyl-CoA → acetate + ATP)	Acetate + ethanol (via acetaldehyde disproportionation);acetyl-CoA may enter Wood–Ljungdahl pathway; acetaldehyde sequestered in bacterial microcompartments (Pdu MCP)	*Acetobacterium woodii*; *Clostridium glycolicum*; Anaerobic consortia (e.g., *Pelobacter* spp., methanogenic co-cultures)

“→” indicates the direction of conversion, while “⇌” indicates a reversible reaction or equilibrium.

## Data Availability

No new data were created or analyzed in this study. Data sharing is not applicable to this article.
